# Virtual screening and docking of lead like molecules against Glutathione-S-Transferase protein from Brugia malayi

**DOI:** 10.6026/97320630014554

**Published:** 2018-12-31

**Authors:** Siva Prasad Venkata Satya Chekkara, Priya Ranjan Kumar

**Affiliations:** 1 Department of Biotechnology, IMS Engineering College, Ghaziabad, Uttar Pradesh-201009, India

**Keywords:** Glutathione-S-transferase, * Brugia malayi*, Virtual screening, Pyrrolidinedione family, Bezimidazole

## Abstract

Glutathione-S-transferase(s) (GST) is an important chemotherapeutic target in lymphatic filarasis caused by *Brugia malayi* and *Wuchereria bancrofti*. It has been playing an important role as major detoxification enzyme and help in intracellular transportation of hydrophobic substrates. Therefore, it is of interest to screen GST from Brugia malayi with millions of known ligands at the ZINC database using AUTODOCK for the identification of potential inhibitors with improved binding characteristics. We report two potent inhibitors ZINC00179016 and ZINC08385519 which are the molecules of pyrrolidinedione and benzimidazole families respectively as potential inhibitors of GST from Brugia malayi with suitable binding properties.

## Background

Filariasis is a parasitic nematode borne tropical and sub-tropical disease. Human filarial nematodes have already affected over 120 million people worldwide with around 1.3 billion people at risk in about 83 countries; it leads to some most debilitating tropical diseases, including Elephantiasis and Onchocerciasis 1. These nematodes cause trouble not only for humans but also live stock. Species like Brugia malayi and Wuchereria bancrofti are the most common causes of the disease in human lymphatic system 2. Geographically the people living in Asia and Africa are mostly affected by this disease due to unhygienic living and sanitary conditions. This mosquito borne disease has been targeted by the WHO for elimination by 2020 3.

The life cycle of the microfilariae Brugia malayi like any other filarial parasite is divided into five different stages. Each stage is divided by four molts and in each of these stages the filarial parasite is differentiated morphologically to different forms in two different hosts, i.e. vertebrates and mosquitoes 2, 4 . The four molts of the micro filarial life cycle the first two are carried out in the mosquito where they grow to infectious L3 stage, after attaining the L3 stage the nematode gets injected into the human host where it grows into adults and completes the other two molts over few months. After fertilization the adult female nematodes releases a large number of the microfilariae into the blood stream from where after a mosquito bite they re-enter the host and their life cycle will be completed 4 .

With the advance knowledge in the filarial biochemistry, bioinformatics and comparative genomics, new potential drug targets have come into light 5,6 , GST is an important chemotherapeutic targets in lymphatic filarasis, it mainly act as critical anti-oxidant and detoxifying agent that is responsible for their survival in the human host 7. GST also provides defense against electrophilic and oxidative damages to nematode tissues which are involved in intercellular transportation of hydrophobic substrates 4. GST is a homodimer of a monomer with 208 residues long and divided into two domains, a small and a larger domain a/� and a respectively 8. GST is also found in humans (PDB Id: 19GS) but it is structurally different from the nematode protein (PDB ID: 5D73) with RMSD value 1.11 9,10, Hence, GST has been selected as apotential drug target in Brugia malayi.

## Methodology

### Protein Homology:

**Figure 1:**
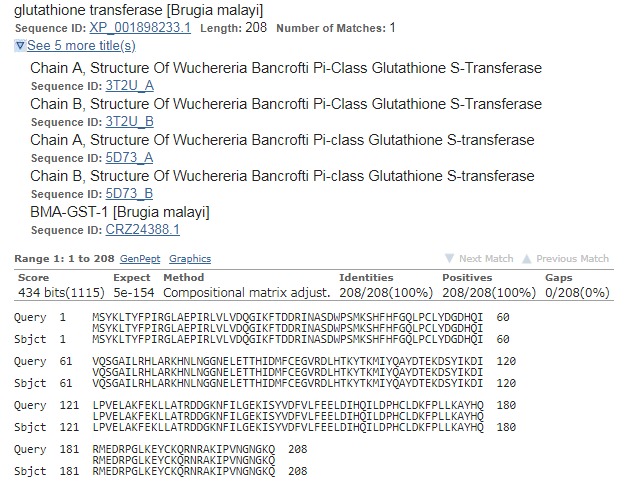
Sequence alignment between query GST Protein with subject sequence identified through blastp and its corresponding PDB Id

The GST protein sequence of Brugia malayi was downloaded from NCBI protein database (Accession no. XP_001898233.1), comprising of 208 amino acids 11, 12. By using BLASTp a 100% similar structure of Wucheria bancrofti was extracted from the protein data bank (PDB ID: 5D73_A). The pdb file with 3D coordinates was downloaded from Protein DataBank 13. The sequence alignment between query and subject with identified PDB id is shown in Figure 1.

### Protein Preparation:

The active site of GST protein was predicted by LigPlot 14 and CASTp 15 . LigPlot is a web basesd application that detects binding sites and pockets in the protein structure, while CASTp is a tool used in the study of protein and its surface topography to detect, locate and measure pockets and voids on the 3D structure of the protein 16 . The protein structure was prepared as a receptor for ligands using the Open Eye software "Make Receptor". With the help of this tool, molecular cavities of the protein were detected. Further, receptor site was selected and put in box of dimensions 15.89 Å x 22.87 Å x 32.28 Å and a total volume of 11732 �3. The balanced shape for the receptor site was generated by defining the inner and outer contours.

### Ligand Preparation:

Structures of 5384 lead like molecules which are analogs of Albendazole and Diethylcarbamazine (commercial molecules) with molecular weight ranging 35 to 350 and the xlogP between -4 to 3.5 were downloaded from the Zinc database in mol2 format to be docked against GST. ZINC is a free database of purchasable compounds that allows us to download a molecule in various file formats 17.

### Screening:

The ligands were screened with high dock resolution against the receptor molecule using FRED. FRED is a docking module of Open Eye software that uses only the protein target structure for pose prediction and scoring, it utilizes the exhaustive search algorithm 18. The top 50 molecules were taken into account as the best docked molecules for the receptor and were again rescored with high optimization and true sort poses.

Gaussian potential indicates the integrity of the ligand poses within the active site of the receptor molecule. Chemgauss4 scoring functions recognizes the shape and hydrogen bond interactions with the protein, while the chemgauss4 is an improved later version that recognizes hydrogen bond geometry with hydrogen bond networks. The FRED 3.0 scoring is based on chemgauss4 scoring pattern and the results with the lowest chemgauss4 score are considered as the best docked molecules with possibility of being used as drugs in the future 19.

### Docking:

The top five molecules obtained from virtual screening were docked on the GST protein active site, using the docking tool Auto Dock 4.0. In process software removes all the water molecules, cofactors and ligands from the protein structure and checks the macromolecule for the polar hydrogens and assigns atomic Kollman charges and atomic solvation parameters. Torsion bonds of the ligands were selected and defined. To evaluate the binding energy of the macromolecule coordinate, a three dimensional grid box of 60 �3 with spacing of 0.3 � was created using Auto Grid which calculated the grid map representing the bound ligand in the actual target docking site 18.

### Validation:

The validation of the results were done by comparing the docking energies of two commercially available drugs Diethyl carbamazine Citrate [N,N-diethyl-4-methylpiperazine-1-carboxamide] and Albendazol [N-(6-propylsulfanyl-1H-benzimidazol-2-yl)carbamate] with the top five ligands obtained from screening. For a given macromolecule-ligand pair the docking energyis comprised of intermolecular interaction energies which includes internal steric energy, hydrogen bond interaction energy, van-der-Waals forces and columbic electrostatic energy of the ligand 19. The receptorligand complex with the lowest binding energy is considered to be the best.

## Results

### Active site prediction:

The protein structure of GST was taken and its active site and druggability was detected, it was then prepared as a receptor site by defining its inner and outer contours. The active site obtained by "Make Receptor", LigPlot and CASTp was compared to the active site taken into account in previous studies. The residues like GLU, ASN, LEU, CYS, VAL, ALA, ARG, TYR, PRO, PHE, THR, HIS were found common in the above mentioned methodoligies and in ealier reported literature 9 ( Table 1).

### Virtual screening:

**Figure 2:**
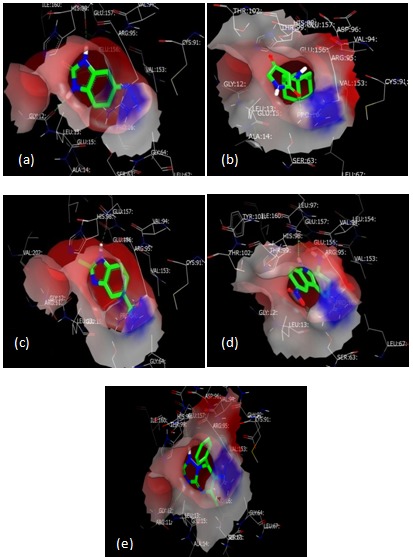
FRED docked structures of top five molecules (a)ZINC08385519 (b) ZINC00179016 (c) ZINC19335442 (d) ZINC00208549(e) ZINC13124456.

Zinc library portion with lead like molecules was firstly screened by using FRED module of Open eye software against GST receptor molecule. The results obtained are mentioned in Table 2 in accordance to their chemgauss4 scores. The above table shows the docking scores obtained after virtual screening using FRED. The docking scores of ten lead like molecules are in comparission with the commercially available drugs, Albendazol and Diethylcarbamazine. The Chemgauss4 score of all the lead like molecules are ranging between -9.50 to - 11.37. Further, the molecules having Chemgauss4 score smaller than -10.00 were selected for docking using Autodock. [ZINC08385519] 5-azido1,3-dihydro-2H-benzimidazol-2-one [Figure 2a], is found to be ranked first lead-like compound out of 5384 selected compounds, its molecular weight is 175.1, chemgauss4 score is -11.37and it is 98% better as compared to the other selected molecules. Its hydrogen bond energy is -7.32KJ/mol. [ZINC00179016] 3-[(1-adamantylamino)methylene]-2,4- pyrrolidinedione [Figure 2b], is found to be second best docked molecule with a chemgauss4 sore of -10.72, its molecular weight is 261.3 with -2.25KJ/mol hydrogen bond energy, and according to the docking report it is 93% better than the other molecules. [ZINC19335442] 2-methyl-1H-benzimidazole-5-carboxylic acid [Figure 2c] is the third best molecule with chemgauss4 score- 10.58, molecular weight 175.2 with -7.16KJ/mol hydrogen bond energy, and is 88% better compared to the other molecules. Molecule scoring the fourth place is [ZINC00208549] 6-nitro-2,3,4,9- tetrahydro-1H-carbazol-1-amine [Figure 2d], with molecular weight 232.3 and hydrogen bond energy -8.07KJ/mol. Its chemgauss4 score -10.32 and it is 83% better compared to others molecules. [ZINC13124456] (E)-5-methyl-7-phenyl- [1,2,4]triazolo[1,5-a]pyrimidin-6(7H)-one [Figure 2e ], is at fifth place with molecular weight 242.3, hydrogen bond energy 9.14KJ/mol, and chemgauss4 score of -10.07. It is 73% better docked compared to other molecules.

### Docking:

The top five molecules obtained after screening were then docked individually on the receptor GST using the Auto Dock tool. Two commercially available drugs Albendazol [N-(6-propylsulfanyl-1Hbenzimidazol- 2-yl)carbamate] and Diethyl carbamazine Citrate [N,N-diethyl-4-methylpiperazine-1-carboxamide](DEC) were also docked on GST to compare the results in binding energy, ligand efficiency, electrostatic energy and hydrogen bonding. The results obtained are mentioned below in Table 3.

**Figure 3:**
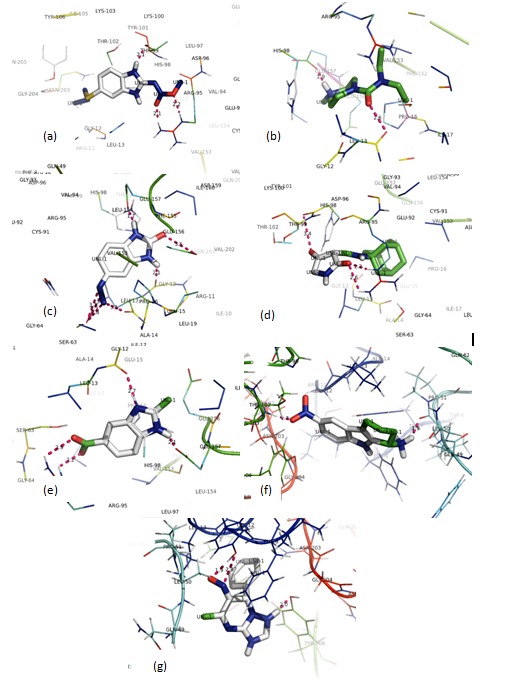
Docked structures of the top five molecules and commercially available drugs Alendazol and DEC on the protein receptor. (a) Albendazol (b) DEC (c) ZINC08385519 (d) ZINC00179016 (e) ZINC19335442 (f) ZINC00208549 (g) ZINC13124456.

While comparing the binding energies of the commercially available drugs Albendazol and DEC to the selected ligands molecules, it has been observed that ZINC00179016 [3-[(1- adamantylamino)methylene]-2,4-pyrrolidinedione] has the lowest binding energy among all. The Figure 3a showes the exact docked structure of Albendazol to the protein receptor. The binding energy of albendazol is -2.84 KJ/mol that is quite high compared to the other molecules and two hydrogen bonds were found in between GST and Albendazol. The docked structure of DEC is shown in Figure 3b , there are no hydrogen bonds in the docked complex because the distance between the ligand and GST residues are greater than the optimal distance required for the hydrogen bond formation. Figure 3c, shows the docked structure of ZINC08385519, which has binding energy -6.18 KJ/mol and form two hydrogen bonds with GST. The binding energy of the ZINC00179016-GST complex is -7.12 KJ/mol which is the lowest energy in the group. This shows better binding affinity of ligand toward receptor. Along with it, one hydrogen bond is also formed in this complex [Figure 3d], gives significant specificity which makes better among all selected compounds. ZINC19335442 binds to the GST protein receptor with the binding energy of -5.94 KJ/mol with a single hydrogen bond as shown in Figure 3e. Figure 3(f) shows the docking of ZINC00208549 against the protein GST with two hydrogen bonds, binding energy of this complex is - 5.27KJ/mol. ZINC13124456 binds to GST to form the receptor ligand complex with the binding energy -3.73 KJ/mol forming two hydrogen bonds as shown in Figure 3g.

## Discussion:

Albendazol and Diethyl carbamazine Citrate are the most common drugs used for the treatment of filarisis. These are being used as a drug since 1980�s, but only these drugs alone are not effective against the adult microfilaria 10, therefore there is an urgent need for competent drugs to overcome these shortcomings. In this work we have done in-silico study of compounds which may possess drug-like properties for the inhibition of microfilariae in the human body. Both Brugia malayi and Wucheria bancrofti are filarial nematodes, and hence the homology of the GST protein is 100% similar. The structure of the Wucheria bancrofti protein glutathione transferase was available online on protein data bank, the PDB structure obtained from the data bank 7. The active site residues detected by CASTp and LigPlot in the protein were similar to the active site residues taken into account in previous studies 7, 9, the same residues were found to be interacting even during present docking studies.

The GST protein receptor was screened against a library of 5384 molecules possessing drug- like properties from the ZINC database. These molecules have the properties similar to drugs and therefore are considered appropriate for futuristic in-silico drug designing and docking studies 18. The Top ten results obtained from the screening were subjected to individual docking against the receptor protein molecule to obtain the exact binding of the protein-ligand complex and their binding energies. The conformation with least binding energy is considered to be the best docked compound. Comparison was done between the binding energies of commercially available Albendazol and DEC drugs with the results obtained in the screening. Diethyl carbamazine citrate (DEC) binds to the receptor GST with binding energy -6.32 kJ/mol without forming any hydrogen bonds because the average distance between the ligand and the receptor is more than the optimal distance required to form hydrogen bond 10. Albendazol is another commercially available drug used for the treatment of filaria, the docking studies of albendazol with the protein receptor shows that the binding energy of the interacting complex is -2.84 KJ/mol and two hydrogen bonds are formed. ZINC00179016 shows a better binding energy (-7.12 KJ/mol) as compared to the clinically approved drugs Albendazol (-2.84 KJ/mol) and DEC (-6.32 KJ/mol). ZINC00179016 with the molecular weight of 260.33 gm/mol and XlogP value 2.2 may be a better drug if clinical studies are carried out on it. ZINC08385519 abenzimidazol compound can also be a good drug for GST receptor to prevent the disease, as benzimidazols are compounds of benzene and imidazol, they are bicyclic, hetrocyclic aromatic compounds with many pharmacological properties like antibacterial, antiviral, anti cancerous and also anti helementhic 20. ZINC08385519 has a good binding energy and is even better in bonding as compared to DEC because it has 2 hydrogen bonds while DEC has none.

## Conclusion

Filaria is a very peremptory disease, if not detected in early stages it becomes incurable for years. It majorly spreads due to the unhygienic living conditions in the tropical and sub tropical regions. Therefore, it is of interest to combat the disease using new drugs with improved efficacy. Hence, we screened the GST from Brugia malayi against selected compounds at the ZINC database using OpenEye and AUTODOCK. The compounds ZINC00179016 and ZINC08385519 with a binding energy of -7.12KJ/mol and - 6.18KJ/mol respectively are showing sigficant results in comaparision with Albendazol and DEC. The binding energy of the above said compounds and the hydrogen bonds indiacates that these may be better inhibitors of GST on par with commercial drugs. It should be noted that further in vitro studies are needed to consider these as potential drug molecules targeting GST of Brugia malayi.

## Figures and Tables

**Table 1:** Comparing active site residues obtained from PDBSUM Ligplot, CASTp and interactive residues reported in literature, the residues highlighted were found to be common in all three.

LigPlot	CASTp	Interactive residues reported in literature
ALA ARG ASN ASP CYS GLN GLU GLY HIS ILE VAL TYR TRP THR SER PRO PHE LYS LEU ASP	ARG GLY LEU PRO ILE SER CYS VAL HIS GLU ASN PHE ASN GLU LYS THR ASP	GLU ASN LEU CYS VAL ALA ARG TYR PRO PHE THR HIS

**Table 2:** Results with ZINC ID, name and chemgauss4 score obtained by FRED.

Sr. No.	ZINC ID	COMMON NAME	CHEMGAUSS4 SCORE
1	ZINC17146904	Albendazol[N-(6-propylsulfanyl-1H-benzimidazol-2-yl)carbamate]	-9.96
2	ZINC00001288	Diethylcarbamazine (N,N-diethyl-4-methylpiperazine-1-carboxamide)	-11.02
3	ZINC08385519	5-azido1,3-dihydro-2H-benzimidazol-2-one	-11.37
4	ZINC00179016	3-[(1-adamantylamino)methylene]-2,4-pyrrolidinedione	-10.71
5	ZINC19335442	2-methyl-1H-benzimidazole-5-carboxylic acid	-10.57
6	ZINC00208549	6-nitro-2,3,4,9-tetrahydro-1H-carbazol-1-amine	-10.32
7	ZINC13124456	(E)-5-methyl-7-phenyl-[1,2,4]triazolo[1,5-a]pyrimidin-6(7H)-one	-10.07
8	ZINC04646972	�N'-bicyclo[3.2.0]hept-2-en-6-ylidenenicotinohydrazide	-9.99
9	ZINC00281407	5,5-diethyl-6-iminodihydro-2,4(1H,3H)-pyrimidinedione	-9.88
10	ZINC00208551	6-nitro-2,3,4,9-tetrahydro-1H-carbazol-1-amine	-9.72
11	ZINC04126512	1-(adamantanylamino)propan-2-ol	-9.54
12	ZINC12651862	(4-Hydroxy-2,6-dimethylpyrimidin-5-yl)acetic acid	-9.5

**Table 3:** Comparison of results between top five screened molecules and albendazol and DEC as reference molecule after docking with Autodock.

Results Molecules	Binding Energy (KJ/mol)	Ligand Efficiency	Electrostatic Energy	Hydrogen Bonds
Albendazol[N-(6-propylsulfanyl-1H-benzimidazol-2-yl)carbamate]	-2.84	0.16	0.61	2
Diethylcarbamazine (N,N-diethyl-4-methylpiperazine-1-carboxamide)	-6.32	0.45	1.41	0
ZINC08385519 (5-azido1,3-dihydro-2H-benzimidazol-2-one)	-6.18	0.48	0.1	2
ZINC00179016 [3-[(1-adamantylamino)methylene]-2,4-pyrrolidinedione]	-7.12	0.37	0.13	1
ZINC19335442 [2-methyl-1H-benzimidazole-5-carboxylic acid]	-5.94	0.46	0.88	1
ZINC00208549 6-nitro-2,3,4,9-tetrahydro-1H-carbazol-1-amine	-5.27	0.31	1.13	2
ZINC13124456(E)-5-methyl-7-phenyl-[1,2,4]triazolo[1,5-a]pyrimidin-6(7H)-one	-3.73	0.21	0.38	2
